# Novel Approach for the Search for Chemical Scaffolds with Activity at Both Acetylcholinesterase and the α7 Nicotinic Acetylcholine Receptor: A Perspective on Scaffolds with Dual Activity for the Treatment of Neurodegenerative Disorders

**DOI:** 10.3390/molecules24030446

**Published:** 2019-01-27

**Authors:** Natalia M. Kowal, Dinesh C. Indurthi, Philip K. Ahring, Mary Chebib, Elin S. Olafsdottir, Thomas Balle

**Affiliations:** 1 Faculty of Pharmaceutical Sciences, School of Health Sciences, University of Iceland, 107 Reykjavik, Iceland; nmp@hi.is (N.M.K.); elinsol@hi.is (E.S.O.); 2 Sydney School of Pharmacy, Faculty of Medicine and Health, The University of Sydney, Sydney, NSW 2006, Australia; dind5627@uni.sydney.edu.au (D.C.I.); philip.ahring@sydney.edu.au (P.K.A.); mary.collins@sydney.edu.au (M.C.); 3 Brain and Mind Centre, The University of Sydney, Camperdown, NSW 2050, Australia

**Keywords:** virtual screening, multi modal, dual mode of action, nAChR, nicotinic acetylcholine receptor, AChE, acetylcholinesterase, docking, lead identification, neurodegenerative disorders

## Abstract

Neurodegenerative disorders, including Alzheimer’s disease, belong to the group of the most difficult and challenging conditions with very limited treatment options. Attempts to find new drugs in most cases fail at the clinical stage. New tactics to develop better drug candidates to manage these diseases are urgently needed. It is evident that better understanding of the neurodegeneration process is required and targeting multiple receptors may be essential. Herein, we present a novel approach, searching for dual active compounds interacting with acetylcholinesterase (AChE) and the α7 nicotinic acetylcholine receptor (nAChR) using computational chemistry methods including homology modelling and high throughput virtual screening. Activities of identified hits were evaluated at the two targets using the colorimetric method of Ellman and two-electrode voltage-clamp electrophysiology, respectively. Out of 87,250 compounds from a ZINC database of natural products and their derivatives, we identified two compounds, **8** and **9**, with dual activity and balanced IC_50_ values of 10 and 5 µM at AChE, and 34 and 14 µM at α7 nAChR, respectively. This is the first report presenting successful use of virtual screening in finding compounds with dual mode of action inhibiting both the AChE enzyme and the α7 nAChR and shows that computational methods can be a valuable tool in the early lead discovery process.

## 1. Introduction

**Figure 1:**
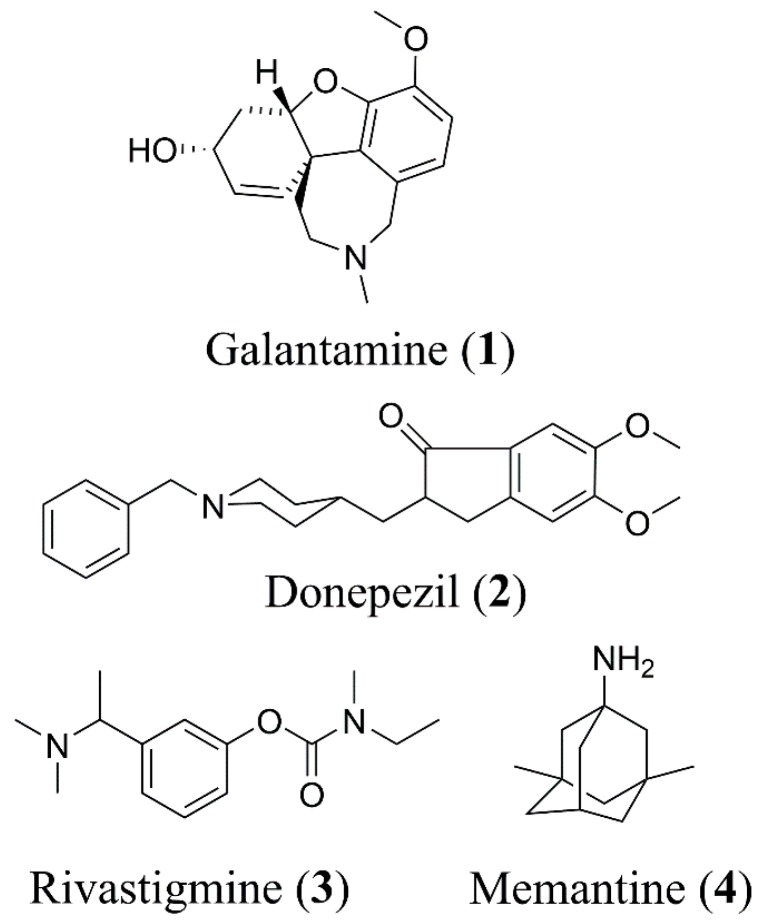
Structures of currently approved Alzheimer’s disease (AD) drugs galantamine (**1**), donepezil (**2**), rivastigmine (**3**) and memantine (**4**).

Alzheimer’s disease (AD) is the most common form of dementia affecting, in particular, the elderly population. So far, the disease is not well understood, and despite huge research efforts by academia and the pharmaceutical industry to understand the origins of the disease, the identification of new drugs to treat or alleviate symptoms of memory impairment have proven difficult. The drugs that are currently on the market are mainly acetylcholinesterase (AChE) inhibitors including galantamine (**1**), donepezil (**2**), and rivastigmine (**3**), and the NMDA receptor antagonist, memantine (**4**) (Figure 1). These drugs do not slow progression of the disease [1], but they help to alleviate some of the symptoms. To further enhance their effect, it has been suggested that combining AChE inhibitors with other “pro-cognitive receptor” drug targets such as NMDA receptors could be a better strategy to alleviate cognitive impairment in dementia. Thus, combining treatments such as AChE inhibitors with memantine could improve outcomes [2,3,4] as reported in clinical trials involving patients with moderate-to-severe AD [5,6]. 

Instead of a poly-pharmaceutical approach where two or more drugs are combined, an attractive alternative would be to have one compound simultaneously targeting multiple receptors [7]. Recently, it was suggested that a multi-target approach might be a viable tactic to combat complex neurodegenerative diseases like AD [8,9]. However, identification of lead molecules for development of multi-modal drugs represents a challenge. 

Computational techniques including virtual screening (VS) are attractive technologies for faster identification of drug leads [8,9] and may also prove to be powerful tools for finding multi-modal drugs, if sufficient structural information of the desired drug targets is available. In the past decade, the number of characterized crystal structures has increased significantly along with developments in docking software and hardware, so it is now possible to filter millions of compounds within a short timeframe. Further, virtual screening against one target proved to be more efficient for identification of drug leads versus conventional high throughput screening (HTS) with success rates of 1–40% versus 0.01–0.14%, respectively [10]. However, dual or multi-target screenings are not common and their success rates differ [11,12]. 

For the treatment of AD, an interesting multi-modal drug profile would be a compound that simultaneously acts as an AChE inhibitor and a positive allosteric modulator (PAM) at one of the pro-cognitive nicotinic acetylcholine receptors (nAChR), especially the α7 or α4β2 subtype. This combination would lead to enhanced synaptic acetylcholine (ACh) levels along with a subtype specific potentiation of nAChRs, which would be a more refined way to modulate the cholinergic system, compared to increasing the dose of an AChE inhibitor alone. In particular, the α7 subtype of the nAChR is an interesting target for AD therapy. Specific activation of this receptor is known to be pro-cognitive [13,14,15,16,17]. 

In the search for new compounds that simultaneously target AChE and the α7 nAChR, we embarked on a lead discovery project using high throughput virtual screening (HTVS). As a model compound we selected galantamine that is marketed as an AD drug and has been described in the literature as a drug with the desired dual mode of action, i.e., inhibitor of the AChE and a PAM at the α7 nAChRs [18,19]. Further, as co-crystal structures with galantamine are available of both AChE [20] and an acetylcholine binding protein (AChBP) [21], a commonly used structural surrogate for nAChRs [22], it seemed to be the obvious choice for a structure guided lead discovery project. 

We developed a protocol for high throughput virtual screening to identify multi-modal drug leads utilizing α7 homology models and an AChE X-ray structure and selected compounds that ranked high in both screens for in vitro evaluation at α7 nAChRs and of the AChE. In the current paper we present the virtual screening results confirming that virtual screening is an attractive technique that can successfully be used to identify compounds simultaneously targeting the AChE and the α7 nAChR.

## 2. Results

In this study we aimed to identify compounds possessing activity at both the α7 nAChRs and AChE. We chose galantamine as our lead compound and performed virtual screening towards homology models of the α7 nAChR and an X-ray structure of an AChE. Compounds that scored well in both screens were subsequently purchased and evaluated by two-electrode voltage-clamp electrophysiology in vitro at the human α7 nAChR expressed in *Xenopus* oocytes and against *Electrophorus electricus* AChE using the Ellman’s colorimetric assay. 

### 2.1. Virtual Screening

We focused the search on natural products and natural product derivatives, which are a valuable source of active compounds against both targets. We screened a database consisting of 87,250 natural products and natural product derivatives from the ZINC database merged with an in-house database containing 250 lycopodium alkaloids. The compounds were virtually screened, first at two homology models of the α7 nAChR, and then the top hits were docked to the active site of a co-crystal structure of the AChE enzyme bound with galantamine. Galantamine was identified amongst the top scoring hits in all screens. After post-docking filtering, 78 compounds were left out, of which a sub-set of 13 compounds (Table 1), i.e., galantamine analogues (**6**, **7**) and structurally unrelated compounds (**8**–**18**), were selected and purchased for biological assessment. 

### 2.2. Assessment of Activity with AChE and α7 nAChRs

The selected compounds and galantamine were initially evaluated for activity with AChE and the α7 nAChR using a single concentration of a compound. For assessment of AChE inhibitory activity, we used a colorimetric microplate assay based on the colour change during reaction of ACh with Ellman’s reagent. All compounds were evaluated at 100 μM concentration and all selected compounds, except compound **13** that was inactive, showed inhibition ranging from 7 to 95.4%. Compounds **6** and **7**, sanguinine and norgalantamine, which are galantamine analogues, showed 96.4 and 96.5% inhibition of AChE activity at 100 µM, respectively. Two additional compounds showed inhibition greater than 70%. These compounds, **8** and **9**, which are structurally unrelated to galantamine, at 100 µM inhibited AChE by 78.5 and 88.1%, respectively. Galantamine (10 µM), the known reference compound, inhibited AChE activity by 91.5%. At a similar concentration (10 µM), compounds **6** and **7** showed 95.5 and 88.6% AChE inhibition, respectively, reaching a plateau at 10 µM. 

**Figure 2:**
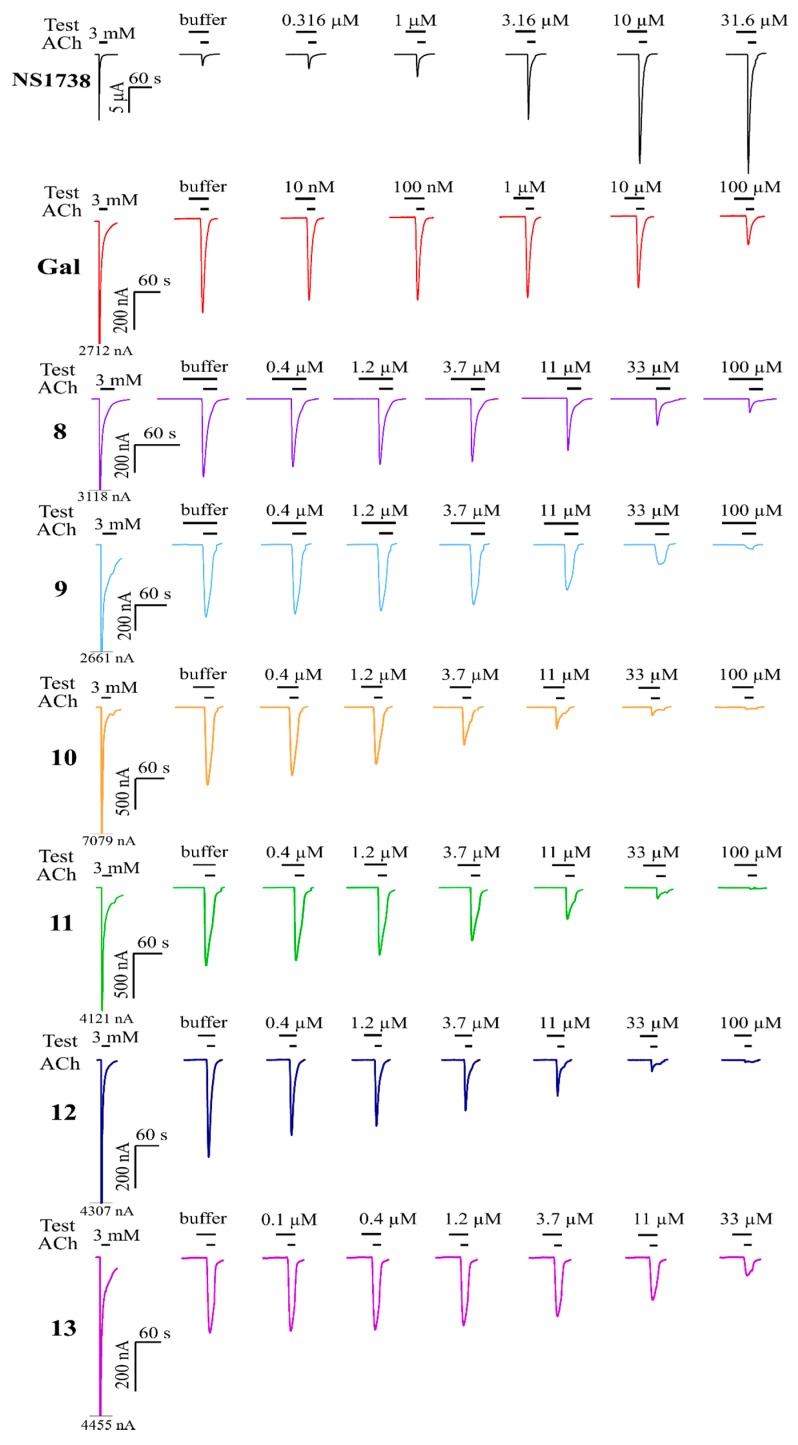
Representative current traces for NS1738, galantamine, and selected compounds from α7 nAChRs expressed in *Xenopus* oocytes. Cells were subjected to two-electrode voltage-clamp electrophysiology experiments; the oocyte membrane potential was clamped at −60 mV. All experiments involved a pre-incubation protocol that consisted of 25 s application of the test solution (or a saline solution for the reference trace) followed by 20 s co-application with 30 µM ACh. The representative traces were baseline subtracted, and the bars above each trace represent the application periods, and concentrations of the test solutions appear above the bars. The majority of the washing periods (3 min) between each trace are omitted.

All compounds were subsequently evaluated at the human α7 nAChR expressed in *Xenopus* oocytes using two-electrode voltage-clamp electrophysiology. The oocytes were pre-incubated with the test compound and subsequently the test compound was co-applied with an EC_20_ concentration of ACh, an experimental design adopted to facilitate identification of PAMs. We first confirmed the ability to identify PAMs by testing NS1738, a well-established α7 PAM [16]. As evident from Figure 2, robust potentiation (440% at 31.6 μM) of (30 µM) ACh-evoked currents was observed. Unexpectedly, galantamine did not show any PAM activity at concentrations ranging from 10 nM to 100 μM, instead, inhibition of the (30 µM) ACh-evoked response was observed. At the highest concentration, galantamine inhibited ACh by 67.3%. These results triggered an in depth evaluation of galantamine effects at the α7 and the α4β2 nAChRs. The outcome of this study was published recently [23], with the conclusion that galantamine is not a PAM of the investigated nAChRs. Out of the 13 tested compounds in the present study, all except compound **18** inhibited the α7 nAChR and compounds **9**–**13** showed more than 90% inhibition at 100 μM. 

**Figure 3:**
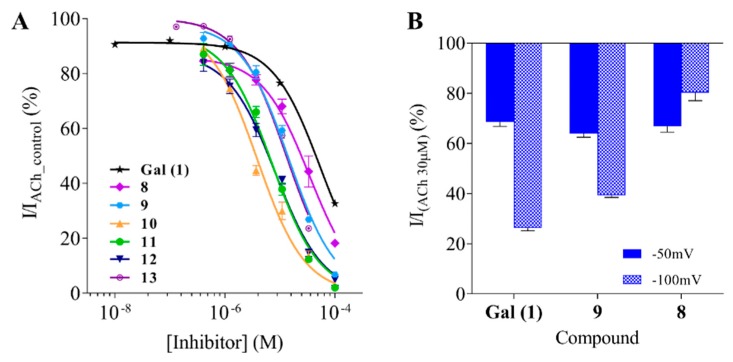
Inhibition of ACh-evoked currents from the human α7 nAChR expressed in *Xenopus* oocytes of galantamine and selected VS hits. (**A**) Concentration-response relationships measured by whole-cell, voltage-clamp experiments (holding potential −60 mV). Inward nicotinic currents were recorded after 25 s incubation with a test or Gal solution followed by 20 s co-application with 30 µM ACh. Peak current amplitudes were measured and normalized with respect to the amplitude of current elicited by 30 µM ACh, *n* = 3. (**B**) Potential-inhibition dependence was determined by conducting experiments at two holding potentials −50 and −100 mV. Oocytes were pre-incubated with the test solution (25 s) and the test solution with 30 µM ACh was co-applied. Gal was tested at 30 µM, compounds 8 and 9 at 10 µM. Peak current amplitudes were normalized with respect to the amplitude of current elicited by 30 µM ACh, *n* = 3–4 cells.

For compounds exceeding 70% and 90% inhibition at 100 μM at AChE and the α7 nAChR, respectively, IC_50_ values were determined based on full concentration response relationships (Table 1, Figure 2 and Figure 3A). To verify the AChE assay, we first tested physostigmine (**5**) and galantamine inhibition and found IC_50_ values of 0.78 and 0.68 μM, respectively, which are in agreement with the previously published results [24,25,26,27]. Two galantamine analogues, **6** and **7**, were ca 3-fold more potent than galantamine itself at the AChE with IC_50_ values of 0.28 and 0.23 µM, respectively. Compounds **8** and **9** were ca 13- and 6-fold less potent compared to galantamine, with IC_50_ values of 10.6 and 5.0 µM, respectively. All compounds for which IC_50_ values were determined at the α7 nAChR (**8**–**13**) were stronger inhibitors than galantamine, with compounds **10**–**12** displaying single digit micromolar potencies corresponding to 6- to 14-fold higher potency. Compound **13** inhibited α7 nAChRs with an IC_50_ value of 13.1 μM. Compounds **8** and **9** were less potent with IC_50_ values of 34.3 and 14.5 μM, respectively. 

Of the 13 screened compounds, two compounds, **8** and **9**, met the criteria for being “dual mode of action”, corresponding to a virtual screening hit rate of 15% across two targets. 

Since galantamine at high concentrations acts as an open channel pore blocker [23], we investigated if the inhibitory activity of the dual mode of action compounds was dependent on membrane potential. We designed a simple experiment where application of a single concentration of a drug was applied at two different membrane holding potentials, −100 and −50 mV. At a lower, more negative potential, the driving force for ion flow is higher and causes a stronger inhibition of ACh induced currents for positively charged channel blockers. Currents elicited in the presence of antagonists (compounds binding elsewhere on the receptor) are independent of the membrane potential. 

In this assay, galantamine as well as compounds **8** and **9** showed ~30% inhibition of (30 µM) ACh-induced currents when tested at −50 mV (note that galantamine was tested at 30 µM and compounds **8** and **9** due to their higher potency, at 10 µM). Inhibition increased for galantamine and compound **9** when tested at −100 mV and was different for each compound: 42% for galantamine and 25% for compound **9.** Inhibition decreased 13% for compound **8** (Figure 3B). These results indicate that compound **8** is binding in the ACh pocket; however, compound **9** might work as a pore blocker or possibly has a mixed mechanism of action. 

## 3. Discussion

In the present study we explored the use of HTVS as a tool for identification of lead compounds targeting two biological targets, AChE and the α7 nAChRs as inhibitors and PAMs, respectively. A dual-acting compound working at these two targets would be highly interesting in relation to developing drugs for the treatment of dementias where both targets independently have been shown to effectively enhance neurotransmission and improve cognitive functions [28].

HTVS is a valuable method; however, it is not devoid of challenges, and hit rates vary significantly depending on the target and the quality of the crystal structure used [12,29]. Requiring a hit to have activity at two independent targets is even more challenging, and there are only a few such examples in the literature. For identification of drug leads against AD, an interesting approach was taken by Domínguez et al., where compounds with neuroprotective activity previously observed in mice were investigated against two enzymes: AChE and γ-secretase [29]. In another approach, Lepailleur et al. searched an in-house library of presumed GPCR compounds and looked for additional effects on histamine H_3_ and serotonin 5-HT_4_ receptors [30]. These dual-target screening projects proved to be successful with hit-rates of 12–33%.

We searched for compounds targeting both AChE and the α7 nAChR. These are two structurally and functionally very different biological targets, an enzyme and a ligand gated ion channel. However, through evolution, their binding pockets have become optimised to efficiently recognise the same neurotransmitter, ACh. Both targets contain a characteristic aromatic cassette that efficiently recognises and binds cations, and identification of dual-action compounds towards these two particular targets may, therefore, not be as difficult as could be anticipated. 

**Figure 4:**
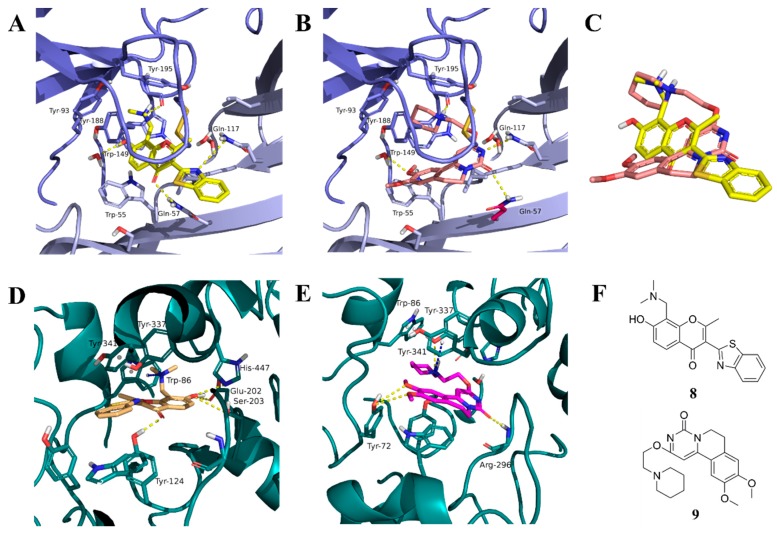
Binding mode of the two dual-active compounds in the α7 nAChR homology models and AChE X-ray structure. Compound **8** in the α7 nAChR (**A**) and AChE (**D**) binding pockets. Compound **9** in the α7 nAChR (**B**) and AChE (**E**) binding pockets. Both compounds superimposed in the α7 nAChR (**C**) pocket showing common pharmacophores. Compound **8** binds to the α7 nAChR (**A**) through three hydrogen bonds (yellow dotted lines) to Q57, Q117, and W149; compound **9** exhibits similar interactions with the same amino acids Q57, Q117, and W149. In the AChE pocket, compound **8** (**D**) binds through hydrogen bonds to Y124, Q202, S203, and H447 and a cation-π interaction to W86 (blue dotted line). Compound **9** (**E**) creates hydrogen bonds to Y72, Y337, and R296 and a cation-π interaction to Y341. (**F**) Structure of compounds **8** and **9**.

The screening approach presented here proves that finding structures with dual activity at the AChE and the α7 nAChR is indeed possible. Most of the compounds identified had some activity at both targets and two met the cut-off criteria of 70% inhibition at AChE and 90% inhibition at the nAChR to merit full evaluation. Interestingly, there appeared to be no correlation between docking scores and measured IC_50_ values. Compounds **8** and **9** were identified as dual-target compounds with a balanced potency at AChE/α7 of 10/34 and 5/14 µM, respectively. Interestingly, the two compounds are not galantamine-like structures, and they differ from each other, but both contain a large aromatic portion and a basic tertiary amine. Their docked poses (Figure 4A,B), which can be superimposed in a way that overlays the protonated nitrogens and aromatic nitrogen heterocycles (Figure 4C), create interactions with Y195 and Q117. This superimposition may define a pharmacophore for compounds targeting both AChE and nAChRs. 

Of the remaining compounds, an additional two, galantamine analogues **6** and **7**, had high potency at the AChE, meaning that 4/13 or 30% of the tested compounds were potent AChE inhibitors. At the α7 nAChR, the individual target HTVS hit rate was higher, 6/13 or 46%, which is not surprising as the VS was biased towards nAChRs in the first place in that only compounds from the top scoring list for nAChR were virtually screened against the AChE. 

In addition to the two dual action inhibitors **8** and **9**, four other compounds (**10**–**13**) were identified as potent α7 nAChR antagonists. Their nicotinic activity has not been previously described. Compounds **8**, **9**, and **12** are new ligands without any previously known biological activities. Compounds **10** and **13** are clinically used drugs. Compound **10** is a well-known antipsychotic drug, cyamemazine, which has anxiolytic properties and is used in the treatment of schizophrenia. It is known to bind to dopamine, serotonin, muscarinic, and histamine receptors [31] but activity at nAChRs has not been reported. The α7 nAChR is considered a target for the treatment of schizophrenia. However, only agonists or PAMs are expected to produce cognition enhancement [32,33]. Compound **13**, nefopam, is another clinically used drug that appeared as an α7 antagonist in our screening. It is a centrally-acting, non-opioid analgesic used for the relief of moderate to severe pain. Its mechanism of action is thought to be by inhibition of serotonin, dopamine, and noradrenaline reuptake, but is not well understood [34]. Again, the α7 nAChR is considered as a target in non-opioid pain treatment with interest in compounds positively affecting the receptor [35]. Compound **11**, flazalone, is known for its anti-inflammatory properties, but has no clinical use [36]. For the above clinically used drugs, the antagonism of α7 nAChRs is unlikely to be linked to the desired clinical effect of the drugs, as in all cases, activation would be required; however, blockade of α7 nAChRs may be linked to side effects. Durrieu et al. [34] studied side effects of nefopam (**13**) and reported occurrence of serious, unexpected neuropsychiatric side effects like confusion and hallucinations. These were correlated to high doses of the drug, which is consistent with its micromolar inhibition of α7 nAChRs described here.

## 4. Materials and Methods

### 4.1. Materials

Plant origin galantamine hydrobromide analytical standard was purchased from PhytoLab GmbH & Co., KG (Vestenbergsgreuth, Germany). Screened compounds were purchased from Ambinter (c/o Greenpharma, Orléans, France). Restriction enzymes were from New England Bio Labs Inc. (Ipswich, MA, USA), and DNA and RNA purification kits were from QIAGEN N.V. (Venlo, The Netherlands). The mMessage mMachine T7 transcription kit was from ThermoFisher Scientific (Waltham, MA, USA). Acetylcholinesterase from *Electrophorus electricus*, acetylcholine chloride, acetylthiocholine iodide, 5,5-dithiobis-(2-nitro-benzoic acid), bovine serum albumin, kanamycin, theophylline, tricaine, collagenase, HEPES, Trizma, salts, and other chemicals not mentioned specifically were purchased from Sigma-Aldrich Co., LLC (St. Louis, MI, USA) and were of analytical grade. 

### 4.2. Homology Modeling and Preparation of Protein Structures

**Figure 5:**
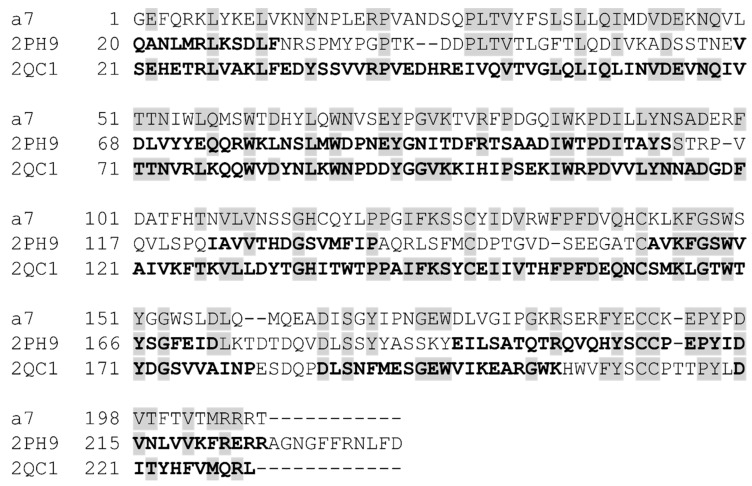
Sequence alignment. Sequence of the human α7 nAChR and templates, AChBP co-crystalized with galantamine (PDB ID: 2PH9), and rat α1 subunit (PDB ID: 2QC1) were aligned using T-Coffee and used to build the homology model. Residues from both templates used in homology modelling are shown in bold. Conserved residues are marked with grey boxes. The sequence identities counting only the parts of the templates that were used for modelling constitute 30 and 39%, respectively, for 2PH9 and 2QC1. For the human α7 nAChR, the sequence was used without the signal peptide.

**Figure 6:**
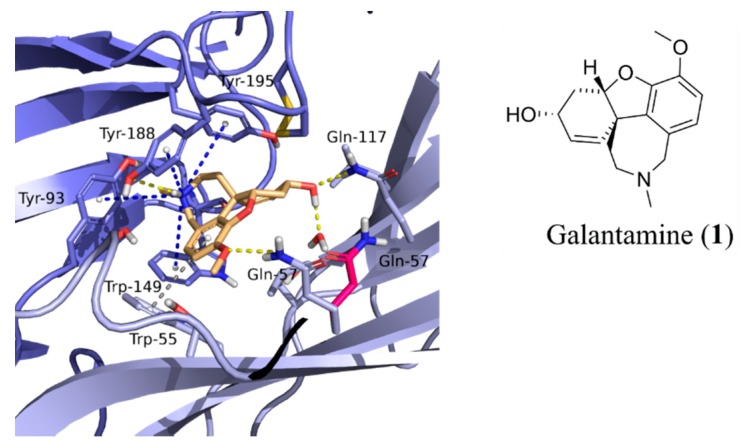
Interactions of galantamine (orange) in the binding pocket of homology models of the α7 nAChR. Interacting residues are shown as purple sticks. Q57 in alternative rotameric state is shown in magenta. Galantamine creates three hydrogen bonds with Q57, Q117, and Y188 (yellow dotted lines) along with several aromatic contacts with the principal receptors: Y93, W149, Y188, and Y195 (blue dotted lines) and to W55 on the complementary side (grey dotted line).

The sequence of the human α7 nAChR was downloaded from the UniProt database (entry P36544, The UniProt Consortium, 2011, www.uniprot.org) and the signal peptide (residues 1–22) and the transmembrane domain (residues 231–490) were deleted. Template structures *Aplysia californica* AChBP with galantamine bound (PDB ID: 2PH9 [21]) and the *mus musculus* α1 nAChR extracellular subunit (PDB ID: 2QC1 [37]) were downloaded from the Protein Data Bank (www.rcsb.org, [38]). After removal of unwanted ligands and heteroatoms, the sequences were initially aligned in T-Coffee [39], and the alignment was subsequently edited according to the following considerations: both templates were used in areas where the two templates were structurally similar, otherwise, 2QC1 was the template of choice, except for regions where this template was distorted due to introduced mutations and bound antagonists. A detailed alignment highlighting the choice of template for specific regions is shown in Figure 5. Homology models with galantamine included in the interfacial binding sites were constructed with MODELLER v.9.9 (Sali-Lab, San-Francisco, CA, USA) [40]. One hundred models were generated and sorted according to DOPE scores [41]. After visual inspection, the model with the most favourable DOPE score was selected and modified as follows: two water molecules (HOH 314 and 315) and a third bridging water molecule (HOH 83) were copied across from the 2PH9 and 2QC1 templates, respectively. Subsequently, the model was energy minimized in Macro Model (Schrödinger Release 2012-1: Macro Model, Schrödinger, LLC, New York, NY, USA, 2017) using the OPLS_2005 force field [42] and the GB/SA water solvation model, after preparation of the structure using the Protein Preparation workflow in Schrodinger’s MAESTRO where hydrogen atoms were added and oriented in an energetically favourable manner followed by protonation/deprotonation of acidic/basic residues and a constrained energy minimization [43]. The water molecules were deemed important to include to block part of the classical agonist pharmacophore to preclude prototypical nicotine-like agonists from the top of the scoring list, which were not of interest in this study. Finally, given that the experimentally determined binding mode of galantamine in the X-ray structure is ambiguous [21], a series of 20 docking runs using the Schrodinger Induced Fit Docking Protocol [44,45] were performed in which protein side chains were sampled and backbone atoms were allowed to adapt in vicinity (10 Å) of the docked ligand. After confirming that no outliers in the Ramachandran plot were present in the vicinity of the binding sites, two top ranked models that differed only by the rotameric state of Q57 were selected as targets for docking. In these structures, galantamine forms cation-π interactions with residues on the principle side of the interface (Y93, Y188, Y195, and W149) and a hydrogen bond to Y188. On the complementary side, galantamine is anchored via a π-π interaction with W55 and hydrogen bonds to a water molecule, Q117, and when in the rotameric state facing the binding pocket, also to Q57 (Figure 6). 

For VS in the AChE enzyme, an X-ray structure of human recombinant AChE co-crystalized with galantamine (PDB ID: 4EY6 [20]) was downloaded from the PDB database and prepared for virtual screening as follows: water molecules were removed except HOH 860, which bridges from galantamine to the receptor (S203 and G122). The X-ray structure was subsequently prepared using the Protein Preparation workflow [43].

### 4.3. Preparation of Databases

A subset of the ZINC database (ZINC version 12, subset Znp98) containing 210,000 natural products and natural product derivatives was downloaded and merged with an in-house database containing lycopodium alkaloid structures (~250 compounds, one low energy conformation per compound). All structures were protonated according to pH 7.4. After calculation of molecular properties with QikProp (Schrödinger Release 2018-4: QikProp, New York, NY, USA) [46], compounds that were not compliant with Lipinski’s rule of five [47], contained reactive or toxic groups, or contained more than one formal charge were discarded. Post filtering, 87,250 compounds remained for high throughput virtual screening.

### 4.4. HTVS and Hits Selection 

**Figure 7:**
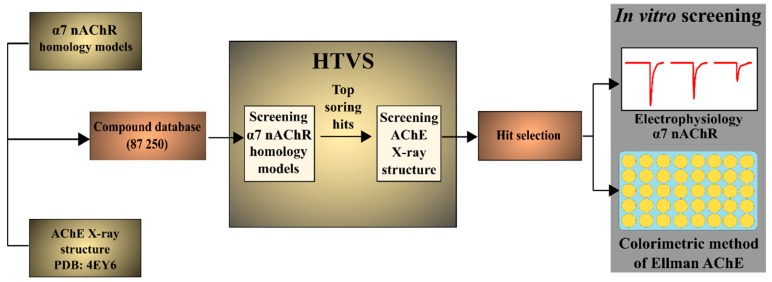
Screening workflow. Virtual screening was performed at two α7 nAChR homology models having different volumes of the binding pocket. Top scoring hits from both models were subsequently screened at the X-ray structure of AChE (PDB ID: 4EY6). From hits scoring high at both targets, 13 compounds were selected and tested in vitro. HTVS = high throughput virtual screening.

HTVS was performed on the two α7 nAChR homology models following the Schrödinger VS protocol (Figure 7). Briefly, three levels of screening were performed (HTVS-, Standard-, and Extra Precision docking) with GLIDE (version 5.9, Schrödinger, New York, NY, USA, 2013) [48,49]. Default settings were used, except the number of compounds that were carried forward between stages was 20% instead of the standard 10%. From the last stage of the nAChR screening, the top 50% of compounds from each homology model (1714 compounds per model) were kept and subsequently filtered, applying a 5 kcal/mol conformational energy penalty cut-off and leaving 1018 and 1031 compounds from the two models, respectively. Then, 1607 unique structures were retrieved from the original database and docked to the AChE crystal structure using Extra-Precision (XP) GLIDE docking and filtered by applying the 5 kcal/mol conformational energy penalty cut-off. 

The ranges of docking scores were broad, therefore, hits with docking scores worse than −10 kcal/mol for AChE and −13 kcal/mol for the nAChR were eliminated. Further, a 400 g/mol molecular weight cut-off filter was applied, leading to 78 compounds that were clustered in Canvas [50] based on the Tanimoto score using Daylight’s fingerprints into eight clusters. After visual inspection, 13 compounds, at least one from each cluster, were selected and purchased. Purity of purchased compounds was >90% as stated by the supplier. The chemical structure and docking scores for the selected compounds and reference compounds are shown in Table 1. 

### 4.5. Validation of Activity at nAChRs 

#### 4.5.1. Molecular Biology

Human α7 nACh receptor subunits were cloned and inserted into expression vectors as described previously [16]. Plasmid cDNAs were linearized using a downstream Not I restriction site and purified. cRNA was prepared and capped from the linearized cDNA using the mMessage mMachine T7 transcription kit according to the manufacturer’s protocol. Purified cRNA was aliquoted and stored at a concentration of 0.5 µg·µL^−1^ at −80 °C until further use. 

#### 4.5.2. Expression of nAChR in *Xenopus laevis* Oocytes

*Xenopus laevis* oocytes were obtained as described previously [51], briefly, ovary lobes were removed by surgical incision, sliced into small pieces, and defolliculated by collagenase treatment.

The protocol for this specific study was approved by the Animal Ethics Committee of the University of Sydney (Protocol number: 2013/5915). Stage V and VI oocytes were injected with a total of ~25 ng of cRNA encoding human α7 nACh receptor with RIC3 (in 5:1 ratio), a protein enhancing the expression of the receptor. Injected oocytes were incubated for 2–5 days at 18 °C in a saline solution (96 mM NaCl, 2 mM KCl, 1 mM MgCl_2_, 1.8 mM CaCl_2_, 5 mM HEPES (hemisodium, pH 7.4)) supplemented with 2.5 mM sodium pyruvate, 0.5 mM theophylline, and 50 µM kanamycin.

#### 4.5.3. Oocyte Electrophysiology

ACh and galantamine were initially dissolved in milliQ water as 10 mM stock solutions. Screened compounds were dissolved as a 10 mM stock solution in DMSO. The maximal DMSO concentration in the final dilution did not exceed 1%. This DMSO concentration did not evoke any current from the receptors. Compound dilutions were prepared in a saline solution on the day of the experiment. 

Electrophysiological recordings from *Xenopus laevis* oocytes were performed using the two-electrode voltage-clamp technique as described previously [23,51]. Briefly, oocytes were placed in a custom-built recording chamber and continuously perfused with a saline solution. The saline solution contained 115 mM NaCl, 2.5 mM KCl, 1.8 mM BaCl_2_, 10 mM HEPES, and was adjusted to pH 7.4 with NaOH. Pipettes were backfilled with 3 M KCl, and open-pipette resistances ranged from 0.3–1.5 MΩ when submerged in the saline solution. Oocytes were voltage clamped at a holding potential of −60 mV unless otherwise stated using an Axon Geneclamp 500B amplifier (Molecular Devices, San Jose, CA, USA). Rapid solution exchange in the oocyte vicinity (order of a few seconds) was ensured by application through a 1.5 mm diameter capillary tube placed approximately 2 mm from the oocyte as described previously [51]. Solution flow rate through the capillary was 2.0 mL·min^−1^. Experiments were performed as follows: nAChR currents were initially evoked with three ACh_control_ applications (~EC_20_, 30 µM), a maximum efficacious concentration of ACh (EC_100_, 3 mM), followed by three additional ACh_control_ applications. Thereafter, test compounds in increasing concentration were applied following a pre-incubation protocol. This involved pre-incubation with the test compound for ~25 s followed by co-application of the test compound with ACh_control_ for 20 s and a wash period of at least 2 min before the next application. 

Initially, the effect of application of 100 µM of a test compound was evaluated, and for the most potent compounds (inhibition at 100 µM > 90%), full concentration response relationships were determined using six concentrations of test compounds from 0.4–100 µM. A detailed description of the protocol was published recently [23]. For experiments evaluating the magnitude of the response as a function of membrane potential, experiments were conducted at two holding potentials: −50 and −100 mV. These experiments also involved a pre-incubation protocol where, first, oocytes were pre-incubated with the test solution (25 s), and secondly, the test solution was applied together with 30 µM ACh (20 s). Galantamine was tested at 30 µM, all other compounds at 10 µM. Concentrations were selected based on the potency of compounds. Peak current amplitudes were normalized with respect to the amplitude of current elicited by 30 µM ACh. All experiments were conducted in triplicate using oocytes from at least two batches from different frogs. 

### 4.6. Validation of Activity at AChE 

In vitro AChE inhibitory activity was studied using the colorimetric method of Ellman [52] using the AChE enzyme from *Electrophorus electricus*. To a 96-well microplate, test solutions were applied along with 0.2 mg/mL bovine serum albumin, 1 mM 5,5-dithiobis-(2-nitro-benzoic acid), and 0.05 mM acetylthiocholine iodide. The reaction was initiated by addition of 0.20 units/mL AChE enzyme and followed by colorimetric detection performed at 405 nm. Experiments were conducted in triplicate. All compounds were dissolved in methanol (maximum 2% methanol at assay conditions that did not affect the enzyme activity) and screened at 100 μM with physostigmine (**5**) as a positive control. 

For compounds exhibiting more than 70% inhibition of ACh degradation, IC_50_ values were determined using six concentrations within the 20–90% inhibition range. 

### 4.7. Data Analysis

Electrophysiological data were analysed using pClamp 10.2 (Molecular Devices, San Jose, CA, USA). During analysis, traces were baseline subtracted and responses to individual applications quantified as peak-current amplitudes. All fitting and statistical calculations were performed using GraphPad Prism 7 (GraphPad, San Diego, CA, USA). A monophasic Hill equation was used in all the non-linear regression calculations. For evaluation of the inhibitory activity, the percentage of remaining peak-current amplitudes relative to that of the ACh_control_ application was calculated. Data were then fitted with the slope set to 1 and the remaining current amplitude at infinitely high compound concentrations set to 0. AChE inhibition data were analysed using GraphPad Prism 7. Absorption readings from the AChE inhibition assay were plotted versus time and linear regression was performed. From the obtained slopes, percentages of inhibition were calculated, and IC_50_ values were determined from non-linear regression calculations.

## 5. Conclusions

We have shown that HTVS can successfully be used to identify compounds with activity at both AChE and the α7 nAChR. Hit rates obtained at both targets significantly exceeded those generally reported in the literature. The choice of galantamine as a lead compound has biased the screening towards antagonists, since galantamine during the course of the project was shown to be an antagonist rather than a PAM. However, finding compounds interacting with two separate targets presents a success in itself and an achievement from a virtual screening technology perspective. The current project can serve as an encouragement for further, more detailed study of the required interactions and more sophisticated designs of HTVS projects providing dual active structures positively affecting the α7 nAChR and inhibiting AChE.

## Figures and Tables

**Table 1:** Structures and corresponding docking scores and in vitro activities of selected hits and reference compounds. All compounds except references galantamine and physostigmine were initially evaluated at 100 µM. For compounds exceeding 70% inhibition at the AChE and 90% at the α7 nAChR, IC_50_ values were determined.

No	Structure	AChE *G-Score ^a^* % Inhibition *^b^* IC_50_ (µM) (pIC_50_ ± SEM)	α7 nAChR *G-Score ^a^* % Inhibition *^b^* IC_50_ (µM) (pIC_50_ ± SEM)	No	Structure	AChE *G-Score ^a^* % Inhibition *^b^* IC_50_ (µM) (pIC_50_ ± SEM)	α7 nAChR *G-Score ^a^* % Inhibition *^b^* IC_50_ (µM) (pIC_50_ ± SEM)
**5**	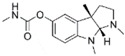 **Physostigmine**	- 91.5 ± 0.3% *^c^* IC_50_ = 0.78 (6.13 ± 0.02)	- - - -	**12**	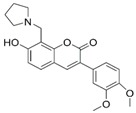	*−11.28* 38.3 ± 4.2% - -	*−14.33* 95.2 ± 2.0% IC_50_ = 8.3 (5.08 ± 0.04)
**1**	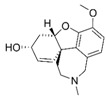	*−11.03* 91.5 ± 0.1% *^d^* IC_50_ = 0.68 (6.14 ± 0.01)	*−15.10* 67.3 ± 0.5% IC_50_ = 54.8 (4.26 ± 0.04)	**13**	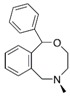 **Nefopam**	*−10.30* 0% - -	*−13.39* 76.3 ± 0.5% *^e^* IC_50_ = 13.1 (4.88 ± 0.02)
**6**	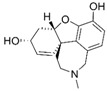	*−11.51* 96.4 ± 0.3% IC_50_ = 0.28 (6.55 ± 0.02)	*−15.10* 45.4 ± 2.3% - -	**14**	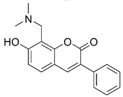	*−12.07* 6.5 ± 1.7% - -	*−13.87* 87.4 ± 1.4% - -
**7**	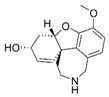	*−10.28* 96.5 ± 0.1% IC_50_ = 0.23 (6.13 ± 0.02)	*−13.05* 64.8 ± 0.7% - -	**15**	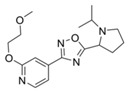	*−11.88* 30.3 ± 4.7% - -	*−13.03* 75.8 ± 0.4% - -
**8**	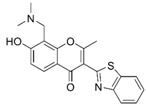	*−13.99* 78.5 ± 0.6% IC_50_ = 10.6 (4.96 ± 0.03)	*−13.03* 81.8 ± 1.3% IC_50_ = 34.3 (4.46 ± 0.05)	**16**	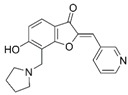	*−10.64* 56.7 ± 4.2% - -	*−13.11* 79.0 ± 1.6% - -
**9**	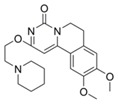	*−10.71* 88.1 ± 0.8% IC_50_ = 5.04 (5.29 ± 0.03)	*−13.11* 93.2 ± 0.3% IC_50_ = 14.5 (4.84 ± 0.04)	**17**	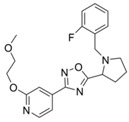	*−12.92* 23.9 ± 5.7% - -	*−14.94* 84.8 ± 0.7% - -
**10**	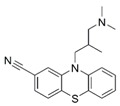 **Cyamemazine**	*−10.35* 32.7 ± 4.2% - -	*−13.27* 97.2 ± 0.5% IC_50_ = 3.9 (5.44 ± 0.05)	**18**	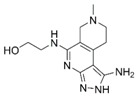	*−11.23* 18.0 ± 8.9% - -	−15.05 2.5 ± 2.5% - -
**11**	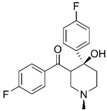 **Flazalone**	*−11.29* 14.0 ± 9.3% - -	*−14.60* 98.0 ± 0.1% IC_50_ = 7.2 (5.14 ± 0.05)				

^a^ Glide G-score (kcal/mol); ^b^ % inhibition at 100 µM; - indicates “value not determined”; ^c^ % inhibition at 5.12 µM; ^d^ % of AChE inhibition at 10 µM; ^e^ inhibition at 33 μM.
